# Management of Extra-Articular and Intra-Articular Distal Radius Malunion

**DOI:** 10.3390/life14091177

**Published:** 2024-09-19

**Authors:** Ting-Yu Liu, Chen-Yuan Yang

**Affiliations:** 1 Department of Orthopedic Surgery, Kuang Tien General Hospital, Taichung 433401, Taiwan; b101098114@hotmail.com; 2 Department of Nursing, Hungkuang University, Taichung 433304, Taiwan

**Keywords:** distal radius fracture, distal radius malunion, extra-articular, intra-articular

## Abstract

This article presents a comprehensive overview of managing extra-articular and intra-articular distal radius malunions (DRM), discussing the pathoanatomy, clinical, and radiologic evaluation, conservative treatment, and surgical strategies. Corrective osteotomy remains the primary surgical intervention for symptomatic DRM. Surgical planning should consider factors such as timing, approach, correction technique, implant, graft, and associated injuries. The correction of extra-articular malunion necessitates brachioradialis tenotomy, circumferential periosteum release, and intrafocal elevation with grafting to facilitate distal radius realignment following osteotomy. Computer-assisted planning with 3D-printed patient-specific instrumentation (PSI) could help execute extra-articular osteotomy with high precision. As for the management of intra-articular malunion, it may require wrist arthrotomy, arthroscopy, or PSI assistance for precise articular osteotomy and reduction of the joint surface. This review highlights the importance of early intervention, thorough preoperative planning, and appropriate surgical techniques to optimize outcomes and minimize complications. Future research should focus on large-scale randomized controlled trials to compare different surgical methods, particularly for intra-articular DRM.

## 1. Introduction

The distal radius fracture comprises approximately 10% of all fracture cases admitted to hospital for surgical intervention [1]. In the past, distal radius malunion (DRM) mostly occurred following conservative treatment. Now, surgical fixation of the distal radius fracture has become more popular, leading to a decrease in the number of malunions after surgical intervention. The incidence of DRM in distal radius fracture is around 24% after conservative treatment with cast, and 11% after surgical intervention. Rates of malunion appear to be lower in operative treatment [2]. DRMs are not actually all symptomatic and noticeable, and the severity of malunion does definitely not correlate with the deformity. However, when DRM becomes symptomatic and limits the activities of daily life, it can lead to considerable disability.

DRM has been defined as a dorsal angulation of ≥10°, ulnar variance of ≥3 mm, and/or radial inclination of ≤15° [3]. But a standard definition of DRM has not yet been established. Other studies in the literature use the criteria of radial inclination <10°, volar or dorsal tilt >20°, radial height 2 mm, and intra-articular step or gap >2 mm [1,4]. DRM may be classified as extra-articular, intra-articular, or combined intra–extra-articular [2,5,6,7]. Extra-articular malunion is still a common complication of the distal radius fracture and can occur in any of the three anatomic planes. In the sagittal plane, fracture displacement can result in an increase in or loss of volar tilt (volar or dorsal angulation). In the axial plane, rotational deformity can happen, and it is suggested that this be approached with computed tomography (CT). Rotational deformity is common with angulated malunions of the distal radius’s displacement; however the amount of rotational deformity does not correlate with the loss of pronation–supination. In the coronal plane, fracture displacement can lead to either an increase in or loss of radial inclination and/or height (ulnar or radial deviation) [7,8]. Intra-articular malunion is also a complication of distal radius fractures. An intra-articular fracture step or gap >2 mm in the wrist joint may lead to intra-articular malunion.

This article offers an in-depth review of the management of extra-articular and intra-articular distal radius malunions (DRMs) by synthesizing findings from extensive searches of PubMed, Embase, and the Cochrane Library. It explores the underlying anatomical changes, as well as clinical and radiological evaluations, and outlines both conservative and various surgical treatment options.

## 2. Pathoanatomy

Although not all misaligned distal radius fractures lead to significant dysfunction, many patients who suffer from DRMs complain about decreasing grip strength, range of motion of the wrist, forearm rotation, and pain with functional impairment. Ali and colleagues mention that patients aged 18 to 65 years who sustain DRM have a worse clinical outcome on the Disabilities of the Arm, Shoulder and Hand (DASH) questionnaire score and the visual analog scale (VAS) pain score but that there were no differences in range of motion or grip strength compared with those patients without malunion [3]. In addition, the long-term complications of DRM such as osteoarthritis (mostly mild) and styloid nonunion have no significant association with DASH scores, VAS pain, satisfaction scores, or grip strength. Many patients are unsatisfied with the bad-looking appearance of the wrist, caused by a protruding ulna head after Colle’s fractures malunion and a bayonet deformity after malunited Smith’s fractures malunion [8].

Malalignment disrupts normal wrist biomechanics, altering load distribution and potentially leading to prearthritic conditions and nerve compression issues. Normal wrist function depends on the anatomical position of the distal radius related to the distal ulna and the carpal bones. The osseous deformity in DRM truly influences the normal function of the radiocarpal joint and produces a limitation of range of motion in extension–flexion arc. Normal wrist range of motion includes a total of 120 degrees of wrist flexion and extension, 50 degrees of wrist ulnar–radial deviation, and 150 degrees of forearm rotation at the distal radioulnar joint. Additionally, the distal radius carries 80% of the axial load through the wrist, while the distal ulna carries 20% of the axial load. Radial deviation of the wrist reduces the load on the ulna, and ulnar deviation increases the load on the ulna [9]. DRM leads to alterations in the biomechanics of the wrist motors. A dorsal angulation of more than 10 degrees significantly affects the movement of the main wrist muscles, while an angulation of more than 30 or 40 degrees greatly changes the moment arms. Similarly, an angulation of more than 10 degrees in the radial inclination has a statistically significant impact on the movement of the main wrist muscles [10,11]. Those alterations have an influence on the distal radioulnar joint (DRUJ) and radiocarpal joint and increase the risk of arthrosis [12,13,14].

Crisco et al. discovered significant alterations in patients with malunited distal radii compared to their uninjured arms. They noted a substantial 25% reduction in the ulnar joint space area, a proximal shift of the centroid by an average of 1.3 mm, and an elongation of the dorsal radioulnar ligament [13]. These alterations play an important role in the dysfunction of forearm rotation. Hirahara et al. concludes that the reduction in distal radius fracture should be within as close to 10 degrees of dorsal angulation as possible to allow patients to perform full rotation of the forearm and wrist [15]. On the other hand, another literature reveals that dorsal angulation up to 30 degrees and radial translation up to 10 mm still have no significant limitation in terms of the pronation or supination of the forearm [16]. Additionally, 5 mm ulnar translation will result in 23% loss of pronation, and 10 mm radial shortening will result in 47% loss of pronation and 29% loss of supination.

In cases of volar angulation, forearm supination is more affected than pronation. Pogue et al. use models compared between the normal state and DRM of varying degrees about contact areas and pressures [17]. Any degree radial shortening increases the total contact area of lunate fossa, especially significant at 2 mm of shortening. With either palmar or dorsal angulating more than 20 degrees in DRM, there is no load distribution change between the scaphoid and lunate areas. But the high-pressure areas were dorsally shifted and more concentrated in the scaphoid and lunate areas. In contrast, decreasing radial inclination shifts the load distribution, so the lunate area increases more load and the scaphoid area decreases.

## 3. Clinical and Radiologic Evaluation

A thorough clinical and radiologic evaluation includes a detailed assessment of the patient’s history, symptoms, functional limitations, and comprehensive physical and radiographic examinations to ensure an accurate diagnosis and optimal treatment outcomes.

Physical examination includes checking for bony prominences or abnormal contours indicative of malunion, palpating the distal radius and ulna for tenderness, and assessing wrist flexion, extension, radial and ulnar deviation, as well as forearm pronation and supination. It also involves determining if there is any impingement during the range of motion; comparing the range of motion and grip strength with the unaffected side; performing a neurological examination, particularly regarding symptoms for carpal tunnel syndrome; and using the Watson test and Ballottement test to assess scapholunate joint and DRUJ instability, respectively. Functional assessment involves evaluating the severity and duration of symptoms like pain, weakness, stiffness, and functional limitations. It includes documenting activities that exacerbate symptoms and using standardized questionnaires such as the DASH score, Modified Mayo Wrist Score, or Patient-Rated Wrist Evaluation (PRWE) score to quantify symptom severity and functional impairment.

A standard series of radiographs of the injured and uninjured wrist is usually performed on all patients suspected of DRM. The posteroanterior and lateral views of the uninjured wrist are often useful for assessing the patient’s natural anatomical characteristics, such as radial inclination, radial length, ulnar variance, and radial tilting. For patients who have undergone previous surgeries, obtaining a series of X-rays from initial fracture and after subsequent surgeries will help in understanding and evaluating the progression of the malunion.

Furthermore, CT scan is often more effective in visualizing the congruity and gapping of the articular surface, as well as in evaluating rotational deformity and detecting degenerative changes and malalignment of the DRUJ. CT with three-dimensional (3D) reconstruction imaging enhances the understanding of the overall malunion pattern and aids in preoperative planning [18,19]. Additionally, for intra-articular malunions, using a subtraction view (removing all distal bony structures including carpal bones, metacarpals, and phalanges) provides a clearer view of the distal radius articular malunion conditions. CT also allows for the creation of precise cutting guides to assist in surgical planning, guiding bone surgeries, and placing internal fixation devices [20]. Magnetic resonance imaging (MRI) can be used to identify soft tissue injuries such as intercarpal ligament and TFCC tears.

## 4. Conservative Treatment

Surgical intervention for patients with DRM is not always necessary. It is important to assess the level of impairment and functional needs of each patient individually. Factors such as age, pain, weakness, function, and loss of mobility should be taken into account when making decisions about whether surgery is needed. It is important to note that in older patients, anatomic deformity and function may not always be directly related to radiography.

Young et al. conducted a retrospective evaluation of 25 inactive, low-activity patients over the age of 60 to assess their functional and radiographic outcomes after receiving a non-surgical treatment for displaced distal radius fracture [21]. The majority of patients (92%) were satisfied with the overall outcome of their treatment, and 88% were able to resume their previous level of activity or work. Even though more than half of the patients had a noticeable clinical deformity in their wrist due to distal radius malunion, none expressed dissatisfaction with its appearance. Overall, most patients were satisfied with the functional outcome, regardless of the radiographic results. Non-surgical treatment for distal radius malunion can lead to a satisfactory outcome, especially for those with low functional demands, and it is also suitable for patients who are not good candidates for surgery [22].

## 5. Surgical Treatment Strategies

### 5.1. General Considerations

#### 5.1.1. Indication

Although there are no definitive surgical indications for DRM [7], corrective osteotomy is the primary intervention for symptomatic DRM, aiming to realign the radius anatomically, thereby enhancing function and alleviating pain. The need for corrective osteotomy is determined by functional limitations, pain severity, DRUJ instability, and esthetic concerns. These symptoms include chronic pain, reduced range of motion, and decreased grip strength due to altered biomechanics. The decision to perform an osteotomy for extra-articular DRM is often based on the patient’s symptoms rather than solely on radiographic findings. Therefore, the noticeable deformity, which leads to dissatisfaction with the wrist’s appearance, should be one of the indications for the procedure. Volar tilt >15° or dorsal tilt > 10° or > 3 mm radial shortening were the suggested surgical indications for the symptomatic extra-articular DRM [23,24]. In cases of intra-articular distal radius malunions, an intra-articular step-off of 2 mm or more indicates the need for corrective osteotomy to prevent long-term cartilage wear and associated osteoarthritis [25].

#### 5.1.2. Contraindication

The contraindications of corrective osteotomy are advanced joint destruction or degeneration, compromised soft tissue conditions, severe soft tissue contractures, fixed carpal malalignment, and patients with significant comorbidities. Correct patient selection is essential to achieve favorable results and minimize the risk of complications from corrective osteotomy.

#### 5.1.3. Timing

Early intervention, typically within 6 weeks to 3 months post-fracture, is often beneficial. At this stage, the callus is still immature, making it easier to correct the deformity and achieve realignment with minimal soft tissue contracture.

Late intervention, occurring three months after the initial fracture, becomes more challenging due to increased soft tissue contractures and the formation of mature callus, which obscures the fracture line boundaries. This stage usually necessitates additional steps, including soft tissue release, mobilization of the malunited fragment, and bone grafting. Research has indicated that early correction is technically less complex and shortens the overall duration of disability when compared to late intervention [26].

#### 5.1.4. Approach

##### Volar Approach

The volar approach has gained popularity due to its several advantages, including better soft tissue coverage and reduced risk of extensor tendon irritation. The extended flexor carpi radialis approach is particularly effective for soft tissue release and malunion correction, allowing for stable fixation with modern locking plates [27]. The volar approach also minimizes the risk of extensor tendon complications, which are more common with dorsal plating. However, potential complications include injury to the median nerve or radial artery and irritation from prominent screws on the dorsal aspect.

##### Dorsal Approach

Traditionally, the dorsal approach has been utilized for dorsally angulated distal radius malunions because, theoretically, this approach facilitates the performance of opening wedge osteotomies and the application of a buttress plate [28]. Through arthrotomy, this approach could also provide direct visualization of the articular surface. Despite its benefits, the dorsal approach is associated with a higher risk of scarring, wrist flexion limitation, and extensor tendon irritation or rupture, even with the use of low-profile plates. Newer plating systems and a better understanding of extensor tendon anatomy have mitigated some of these issues, but the complication rate remains higher compared to the volar approach [29].

##### Combined Volar and Dorsal Approach

For complex cases that involve both intra-articular and extra-articular malunions, a combined volar and dorsal approach may be warranted. This technique allows for a comprehensive correction of deformities across multiple planes, enhancing anatomical restoration. In cases with dorsal intra-articular malunion, the use of a dorsally applied 3D-printed osteotomy jig might be indicated to achieve a precise reduction, followed by fixation with a volar locking plate. However, caution is needed due to potential postoperative complications such as swelling and adhesions arising from the dual approach.

##### Arthroscopy-Assisted Approach

The scope-assisted approach utilizes dorsal portals to assess the condition of intra-articular malunions, thus replacing the role of the traditional dorsal approach with wrist arthrotomy [30]. These techniques offer quicker recovery times and fewer complications associated with dorsal approaches. However, they require high technical proficiency and substantial experience with arthroscopic procedures.

### 5.2. Extra-Articular Malunion

#### 5.2.1. Release of Soft Tissue Contracture

Soft tissue contractures around distal radius malunions present a significant challenge before bony correction. As the duration of injury increases, soft tissue contractures tend to worsen, making attempts to simply correct bony alignment to overcome soft tissue tension and scarring often futile. Extensive release of scar tissue and elevation of the periosteum circumferentially around the malunion site are crucial for achieving correction and lengthening. The dorsal periosteum could be released via an extended flexor carpi radialis approach. A tenotomy of the brachioradialis tendon is usually performed to facilitate radius lengthening after osteotomy.

#### 5.2.2. Corrective Osteotomy

With thorough preoperative planning, corrective osteotomy involves several important surgical steps and considerations to ensure successful outcomes.

##### Creating Osteotomy Plane

To create an osteotomy plane as planned preoperatively, first, multiple K-wires are used to drill holes along the designed cutting plane. Subsequently, a small osteotome and micro saw are used to connect these drilled holes into a line, advancing towards the opposite cortex to complete the osteotomy plane. During the process, fluoroscopy is employed for guidance. Instruments must protect the tendons on the opposite side to prevent iatrogenic tendon rupture.

##### Length

Radius shortening from malunion can be addressed by radius lengthening or ulna shortening. Radius lengthening is preferred for significant radial shortening with complex deformities, offering better functional outcomes but requiring high technical expertise. Ulna shortening, while simpler, is effective mainly for isolated ulnar impaction syndrome, yet it risks complications in the distal radioulnar joint, especially with reverted inclination. A comparative study found that distal radius lengthening osteotomy (DRLO) provided superior pain relief and functional improvement compared to isolated ulna shortening osteotomy (USO). Patients in the DRLO group also showed significantly better postoperative VAS scores and DASH scores. Although USO is quicker and less complex, it has a higher incidence of DRUJ osteoarthritis and often necessitates further interventions [24].

**Figure 1:**
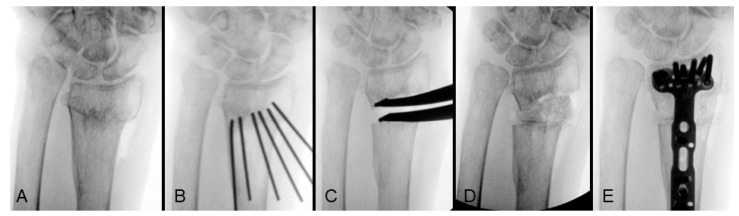
Correction of shortening from extra-articular distal radius malunion: (**A**) preoperative anteroposterior view; (**B**) osteotomy plane created by multiple K-wires; (**C**) length corrected with lamina spreader; (**D**) allograft insertion; (**E**) definitive fixation with volar locking plate. Image courtesy of the corresponding author Chen-Yuan Yang, MD.

To achieve radius lengthening, simple manual traction as treating acute fracture is generally ineffective, even after extensive soft tissue release. The lengthening should be addressed from the osteotomy site using two primary methods: 1. Applying a distraction force with a lamina spreader to incrementally open the gap. 2. Inserting osteotomes into the osteotomy site and gradually spreading them apart to create and widen the gap. This process is monitored by fluoroscopy to ensure proper alignment and height. Once the appropriate radial height is achieved, the osteotomy gap is filled with bone graft to maintain the correction. Allograft or autograft can be used depending on the availability and the extent of the lengthening required. Figure 1 demonstrates the step-by-step process of the length correction for an extra-articular distal radius malunion (Figure 1A). The osteotomy plane was initially formed by multiple K-wires (Figure 1B), followed by radial height lengthening using a lamina spreader (Figure 1C). The defect further was then filled with allograft (Figure 1D), and definitive fixation was achieved with a volar locking plate (Figure 1E). According to the lengthening technique reported by Huang et al., all 10 enrolled patients achieved bone healing within three months post-surgery. The average lengthened distance (change in ulnar variance) was 5 mm, with a range of 3 to 8.5 mm [31].

##### Tilt and Inclination

**Figure 2:**
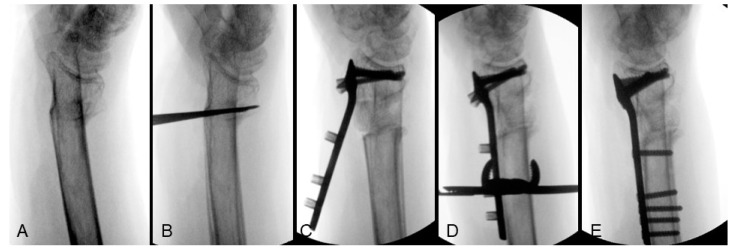
Correction of tilt from extra-articular distal radius malunion: (**A**) preoperative lateral view; (**B**) osteotomy plane created by multiple K-wires; (**C**) allograft insertion and apply the articular locking screws of volar locking plate; (**D**) volar tilt corrected by clamping the plate to the radial shaft; (**E**) definitive fixation. Image courtesy of the corresponding author Chen-Yuan Yang, MD.

In cases with a relatively small osteotomy gap, intrafocal Kapandji K-wires are utilized to facilitate adjustments in alignment. One K-wire is placed dorsally to act as a lever for adjusting the volar tilt, while another intrafocal Kapandji K-wire is positioned radially to correct the radial inclination. However, if greater lengthening is required, resulting in a larger osteotomy gap, the lever effect of the Kapandji K-wire may diminish. In such cases, the distal fragment can be initially secured using the corresponding distal locking screws of a volar locking plate, utilizing the plate as a reduction template. By clamping the shaft end of the plate to the radial shaft, substantial correction of the volar tilt can be achieved even at large angles. Figure 2 illustrates the sequential process of tilt correction for an extra-articular distal radius malunion (Figure 2A). The osteotomy plane was initially formed by multiple K-wires (Figure 2B) and a defect was filled with allograft after radial height lengthening. An anatomical plate was used as a reduction template that the volar tilt was corrected by applying the subchondral articular locking screws first (Figure 2C), then clamping the plate to the shaft to rotate the articular fragment into the correct position (Figure 2D), and finally achieving definitive fixation by inserting all the remaining screws (Figure 2E).

##### Implant

The fixation implant for the osteotomy can be a volar locking plate, dorsal locking plate, or intramedullary nail. Volar locking plates with fixed-angle or variable-angle locking screws are generally preferred due to their strong stability and lower complication rates, especially in common cases with volar tilt loss and radial shortening. Dorsal locking plates are advantageous for direct visualization and correction of dorsal angulation but carry a higher risk of tendon complications [32]. Intramedullary nails offer a minimally invasive option with strong fixation but are technically demanding and less versatile for complex deformities [33].

##### Graft

Allograft is preferred as a grafting material to reduce the disadvantages of autograft, such as donor site morbidity and limited quantity, which may be insufficient for large defects [31]. Studies report that allografts and autografts have comparable healing capabilities in the surgical correction of distal radius malunion [34].

Although some of the literature indicates that no graft is needed to fill the void at the osteotomy site and union can still occur without issues [35,36], the importance of allograft extends beyond promoting healing; it plays a crucial role in maintaining basic alignment during the correction process. Whether using structural grafts for larger defects requiring stable mechanical support or non-structural grafts for sequentially filling the incrementally lengthened gap, allografts are essential.

##### Associated Injury Management

If preoperative carpal tunnel syndrome is present due to dorsal tilt, the volar approach can be extended distally to release the transverse carpal ligament. If performing a substantial lengthening of the radius along with correcting a significant tilt deformity, I would suggest adding a dorsal approach for extensor pollicis longus tendon transposition above the extensor retinaculum to reduce excessive tendon tension post-correction and prevent tendon rupture. After completing the bony correction, the stability of the distal radioulnar joint should be reassessed using the Ballottement test. In cases of marked instability, a TFCC foveal repair with a suture anchor or transosseous suture is required.

##### Patient-Specific Instrumentation (PSI)

Using computer-aided design software, surgeons can create virtual models of the distal radius. This technology allows for the simulation of the osteotomy and the subsequent fixation, providing a detailed surgical plan that can be tailored to the patient’s specific anatomy.

PSI involves the use of custom-made guides that are designed based on the patient’s CT scan data. These guides are 3D-printed and help to execute the osteotomy with high precision. They ensure that the cuts are made at the exact planned location and angle, reducing the margin of error. A randomized controlled trial compared the efficacy of 3D computer-assisted preoperative planning to conventional two-dimensional (2D) planning for the corrective osteotomy of distal radius malunion. Radiographic analysis showed that 3D planning significantly improved mean residual volar angulation and radial inclination compared to the conventional approach [37].

### 5.3. Intra-Articular Malunion

Unlike extra-articular malunions, the greatest challenge with intra-articular malunions lies in clearly identifying the pattern of the malunion on the distal radius articular surface while ensuring precision in each step of the osteotomy, reduction, and fixation. Here are the advantages and disadvantages of various methods:

#### 5.3.1. Simple Fluoroscopy

In the earlier literature, there are studies documenting that intra-articular malunion correction was performed using only fluoroscopy, without performing wrist arthrotomy [38]. However, fluoroscopy does not provide a detailed 3D view of the joint surfaces, which may result in suboptimal reduction in complex fractures compared to techniques that offer enhanced visualization. This is because conventional fluoroscopy only provides a rough 2D image of the scaphoid fossa and lunate fossa joint surfaces in the anterior-poster view and lateral views separately, making it quite challenging to achieve a more three-dimensional and precise reduction and fixation.

#### 5.3.2. Wrist Arthrotomy

Wrist arthrotomy is almost performed from the dorsal side to avoid the risk of volar ligament injury and instability that can occur with a volar arthrotomy. To expose the distal articular surface, the dorsal joint capsule is incised along the line of the distal radial articular surface as necessary. It is important to retain at least a 1 mm margin of the capsule on the distal radius to ensure that it can be reattached [39].

Dorsal arthrotomy allows for direct visualization and manipulation of the fracture fragments, which can be critical for achieving precise anatomic reduction. However, arthrotomy is more invasive compared to other techniques, which leads to longer recovery times and an increased risk of complications such as infection, adhesion, extensor tendon rupture, and joint stiffness due to extensive soft tissue dissection [40].

#### 5.3.3. Computer-Assisted Virtual Planning with 3D-Printed Patient-Specific Instrumentation (PSI)

Computer-assisted virtual planning with 3D-printed PSI offers multiple advantages in the surgical correction of distal radius intra-articular malunion. Theoretically, this method allows for highly accurate preoperative planning and execution, which can lead to better anatomical restoration of the fracture. By using advanced imaging and software, surgeons can create custom instruments and guides that fit perfectly to the patient’s unique anatomy. This customization enhances the precision of the osteotomy and fixation, ensuring that the surgical tools align exactly with the planned cuts and placements [20]. Corrective osteotomy using patient-specific instruments could improve postoperative wrist range of motion (ROM), grip strength, VAS for pain, and PRWE scores in patients with intra-articular malunions of the distal radius [41,42]. Additionally, detailed preoperative planning can streamline the operative procedure, reducing the radiation exposure and duration of surgery.

However, the use of computer-assisted virtual planning with 3D-printed PSI also has various disadvantages, including soft tissue damage, limited flexibility for real-time adjustments, high costs, and technological dependence. Unlike pure extra-articular corrective osteotomy, which can often be achieved with a single PSI for alignment correction, intra-articular malunions require multiple PSI jigs. Each jig is employed sequentially for positioning K-wires, predrilling for screws, and performing separate osteotomy planes. The accuracy of the procedure relies on close contact between the jig and bony surfaces or landmarks, which necessitates more extensive soft tissue dissection and stripping in both volar and dorsal wrist approaches. This leads to greater soft tissue damage.

Secondly, the PSI system might lack flexibility for real-time adjustments. During surgery, surgeons must still rely on traditional fluoroscopy or intraoperative CT to confirm changes in the joint surface. However, the former provides only 2D images, while the latter requires waiting for scan times and involves the drawbacks of radiation exposure and the inability to adjust in real time. In the interconnected steps of joint surface correction, it is crucial to carefully evaluate whether everything is proceeding according to plan.

Thirdly, the production and technology behind custom instruments are expensive, potentially limiting their widespread adoption and accessibility. This approach requires advanced imaging, specialized software, and technical expertise, resources that may not be available in all medical centers.

#### 5.3.4. Arthroscopic-Assisted Osteotomy

Wrist arthroscope-assisted surgery provides a precise, real-time adjustable and minimally invasive solution to manage the intra-articular malunion and associated soft tissue injuries simultaneously. One of the primary advantages is the enhanced visualization of the joint, which allows for flexible adjustments and precise reduction of the mobilized bony fragments under direct vision throughout the procedure. This technique is minimally invasive compared to traditional open methods, reducing soft tissue damage and preserving the capsular blood supply, which is crucial for the healing process. Additionally, arthroscopy allows surgeons to identify and address associated intra-articular pathologies like cartilage damage or ligament injuries such as TFCC and scapholunate interosseous ligament during the same procedure [43]. The disadvantage of the wrist scope-assisted technique is that there is a learning curve to familiarize oneself with portal setting and equipment handling, making it less accessible to surgeons who are not specialized in this area.

During the wrist scope-assisted procedure, the intra-articular malunion and associated soft tissue injuries were evaluated via the 3-4/6R/midcarpal radial (MCR)/ midcarpal ulnar (MCU) portals. After confirming that there was no severe cartilage wear on the scaphoid fossa and lunate fossa, and identifying the pattern of the malunion, the osteotomy process could begin. However, if severe cartilage wear is discovered, shifting to a salvage procedure involving arthroscopic partial wrist fusion may be necessary [44,45].

**Figure 3:**
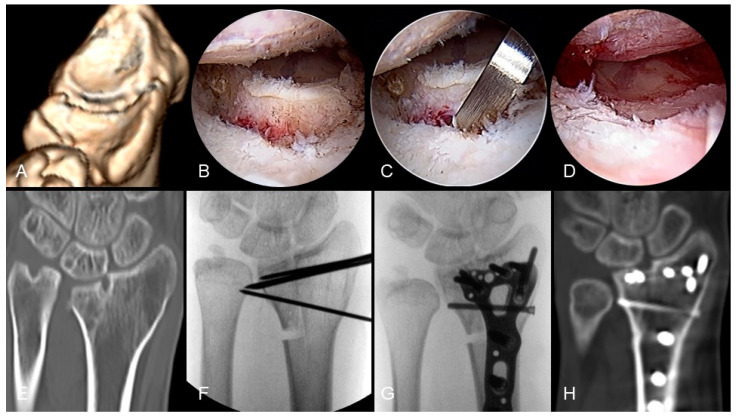
Arthroscope-assisted corrective osteotomy of intra-articular distal radius malunion: (**A**) preoperative articular step-off noted from 3D CT reconstruction with carpal bones subtraction; (**B**) articular step-off noted through arthroscope; (**C**) inside-out osteotomy; (**D**) reduction of intraarticular malunion; (**E**) preoperative CT coronal view; (**F**) temporary K-wires fixation of reduced articular surface; (**G**) definitive fixation and length correction with additional ulna shortening osteotomy; (**H**) follow-up CT coronal view 8 months later. Image courtesy of the corresponding author Chen-Yuan Yang, MD.

First, an inside-out osteotomy was performed: using 3-4 mm straight and curved osteotomes, the step-offs or gaps of the articular surface were gradually pried open. Then, through a volar extended FCR approach, the extra-articular malunion scarring was thoroughly released, followed by an outside-in osteotomy; this could be achieved by using an osteotome with a guiding needle inserted from the joint capsule or an aiming guide with K-wires to connect the intra-articular and extra-articular osteotomies [30]. The goal was to independently mobilize each malunited intra-articular fragment. Subsequently, under the scope, these particular fragments were reduced and transfixed with K-wires and then a locking plate was used for the definitive fixation of both intra-articular and extra-articular malunions. After completing the bony procedure, if concurrent injuries to the TFCC, scapholunate interosseous ligament, or lunotriquetral interosseous ligament are identified, corresponding repair or reconstruction surgeries can be performed simultaneously. Figure 3 shows the progressive steps involved in arthroscope-assisted correction for an intra-articular distal radius malunion. The process begins with a preoperative assessment of the articular step-off from a 3D CT reconstruction, with carpal bones subtracted (Figure 3A), and further verification through the arthroscope (Figure 3B). An inside-out osteotomy is then performed (Figure 3C), followed by the reduction of the intra-articular malunion (Figure 3D). The preoperative condition is also shown in a CT coronal view (Figure 3E). Temporary K-wire fixation stabilizes the reduced articular surface (Figure 3F), and definitive fixation along with length correction is achieved through a distal radius locking plate and additional ulna shortening osteotomy (Figure 3G). An 8-month follow-up CT coronal view confirms the joint congruence and bony union of the corrective osteotomy (Figure 3H).

Del Piñal et al. reported on 11 patients that underwent arthroscopically assisted osteotomy, which allows for a direct visualization of the osteotomy site, resulting in good midterm clinical and radiologic outcomes, including reducing step-offs to 0 mm in most cases [46]. Satria et al. reported on three patients undergoing arthroscopic-assisted intra-articular osteotomy. All patients showed improvement in QuickDASH and PRWE scores, as well as in the ROM of the operated wrist [47].

## 6. Conclusions and Future Directions

To reduce distal radius malunions, accurately diagnosing, properly reducing, and ensuring stable fixation through immobilization or surgical intervention at the onset of an acute fracture are crucial. Extra-articular distal radius malunion correction involves circumferential soft tissue and periosteum release, intrafocal elevation for realignment following osteotomy, stable fixation, and could include the use of patient-specific instrumentation for enhanced precision and outcomes. Intra-articular distal radius malunion management could require wrist arthroscopy or patient-specific jig assistance for precise osteotomy and reduction of the articular surface. Future research could focus on large-scale randomized controlled trials to compare the outcomes of different surgical techniques, particularly patient-specific instrumentation and arthroscopy-assisted osteotomy for intra-articular distal radius malunion.
